# Radiotracers for *in situ* infection imaging: Experimental considerations for *in vitro* microbial uptake of gallium-68-labeled siderophores

**DOI:** 10.1016/j.diagmicrobio.2024.116522

**Published:** 2024-12

**Authors:** Asma Akter, Margaret S. Cooper, Afnan M.F. Darwesh, Robert C. Hider, Philip J. Blower, Nicholas M. Price, Oliver Lyons, Silke Schelenz, Varun Mehra, Vincenzo Abbate

**Affiliations:** a Institute of Pharmaceutical Science, Faculty of Life Science & Medicine, King's College London, London, UK; b School of Biomedical Engineering & Imaging Sciences, Faculty of Life Science & Medicine, King's College London, London, UK; c Department of Radiologic Sciences, Faculty of Applied Medical Sciences, King Abdulaziz University, Jeddah, Saudi Arabia; d Directorate of Infection, Guy's & St Thomas' NHS Foundation Trust, London, UK; e Department of Surgery, University of Otago, Christchurch, New Zealand; f Department of Microbiology, Kings College Hospital NHS Foundation Trust, London, UK; g Department of Hematology, King's College Hospital NHS Foundation Trust, London, UK

**Keywords:** Diagnostics, Infection imaging, ^68^Ga-siderophores, Uptake method, PET tracers, DFO-B

## Abstract

*In vitro* screening of gallium-68(^68^Ga)-siderophores in pathogens relevant to infections is valuable for determining species specificity, their effect on cell viability, and potential clinical applications. As the recognition and internalization of siderophores relies on the presence of receptor- and/or siderophore-binding proteins, the level of uptake can vary between species. Here, we report *in vitro* uptake validation in *Escherichia coli* with its native siderophore, enterobactin (ENT) ([^68^Ga]Ga-ENT), considering different experimental factors. Compared with other reporting methods of uptake, ‘% Added dose/10^9^ CFU/mL (% AD/10^9^ CFU/mL),’ considering the total viable count, showed a better comparison among microbial species. Later, *in vitro* screening with [^68^Ga]Ga-desferrioxamine B (DFO-B) showed high uptake by *Staphylococcus aureus* and *S. epidermidis*; moderate uptake by *Pseudomonas aeruginosa*; poor uptake by *E. coli, Candida albicans*, and *Aspergillus fumigatus*; and no uptake by *Enterococcus faecalis* and *C. glabrata*. Except for *S. epidermidis*, [^68^Ga]Ga-DFO-B did not reduce the cell viability.

## Highlights

• Validation of *in vitro* ^68^Ga-siderophore uptake in *E. coli* and *A. fumigatus.*
• Uptake results expressed as ‘% Added dose/10^9^ CFU/mL’ to compare bacterial and yeast species.
• [^68^Ga]Ga-DFO-B uptake varies among microbial species.
• [^68^Ga]Ga-DFO-B uptake has no significant effect on assessed bacterial and yeast cell viability.

## 1 Introduction

Siderophores are iron chelators produced by most bacteria and fungi (and some plants) to sequester and store iron(III) (Fe^3+^) from the environment for their growth and survival, for instance, from the host during infection [1,2]. Although siderophores are specific to different microbes (characterized by specific siderophore-binding receptors), many bacteria and fungi can also utilize siderophores that are not their own (xenosiderophores) [[3], [4], [5]]. Radiometal gallium-68(III) (^68^Ga^3+^), a Positron emission tomography (PET) tracer, has a chemical resemblance to Fe^3^+ (similar charge, ionic radius and ligand donor preferences), allowing a ‘trojan-horse’ approach, exploiting siderophore-mediated iron transport to deliver ^68^Ga instead of iron into microbial cells to image and localize infection in the body [6]. Both *in vitro* and *in vivo* preclinical studies with ^68^Ga-siderophores from the literature have produced promising results, as they are specific to microbes, stable and sensitive *in vivo* [[7], [8], [9], [10], [11], [12]]. These siderophore-based radiotracers could benefit high-risk individuals, such as those recovering from surgery, immunocompromised individuals due to chemotherapy or transplantation, or those prone to opportunistic, nosocomial, and invasive infections [13]. In addition, imaging techniques that aid in distinguishing between bacterial and fungal pathogens could reduce the turnaround time to prescribe appropriate antimicrobial treatments and improve patient outcomes. Owing to its *in vivo* infection model success in recent literature [14,15], desferrioxamine B (DFO-B) (a clinically approved siderophore for iron overload treatment) with ^68^Ga is being subjected to a small observational study on vascular graft infections (VGEI) in the UK (NCT05285072) [13].

*In vitro* experiments are prerequisites to confirm the selectivity and specificity of a radiotracer for targeted pathogens to evaluate the utility of the radiotracer in *in vivo* small animal infection models. Standardized reporting and reproducible studies across research groups at the preclinical stage are crucial [16]. A literature search revealed variation in the experimental settings in the conduct of *in vitro* uptake assays and in the reporting mode, which makes it difficult to compare studies. Additionally, studies provided less insight into infection-specific considerations, such as the type of infection, pathogens involved, site of infection, average microbial load at the site of relevant infection, growth phases of pathogens, biofilm formation, and experimental considerations, such as removal of any iron contaminants or residual iron from consumables, use of appropriate growth and assay media, and methods of cell preparation. The initial inoculum and viability count after the uptake assay are also important for interpreting the results. The reproducibility of *in vitro* results in variable laboratory settings remains questionable, as the timing and degree of iron depletion/addition are probably also relevant, as they alter transcriptional activity in bacteria [17].

In this study, we aimed to address some of these experimental considerations and performed a series of *in vitro* uptake assays in bacterial and fungal cells. In addition, we evaluated the *in vitro* uptake of [^68^Ga]Ga-DFO-B in both bacterial and fungal cells, including the common pathogens for hospital-acquired, medical implant device-related infections, for instance, VGEI [[18], [19], [20]], and opportunistic infections, including *Escherichia coli, Staphylococcus aureus, S. epidermidis, Pseudomonas aeruginosa, Enterococcus faecalis, Candida albicans, C. glabrata*, and *Aspergillus fumigatus*.

## 2 Materials and methods

### 2.1 Chemicals and siderophores

Unless stated otherwise, all chemicals, reagents, and culture media were purchased from Sigma-Aldrich (UK). Siderophores (Table **S1**) were purchased either from Sigma-Aldrich (UK) or from Biophore Research Products (Germany). Fe-DFO-B and Fe-PVD (cold Fe-siderophores as blocking agents) were prepared using Fe_2_(SO_4_)_3_.5H_2_O or FeCl_3_.6H_2_O with DFO-B and PVD at an equimolar ratio. The complex was incubated at room temperature for 15 min, and the pH was adjusted to 6.5-7.0. To minimize metal contaminants, plastic consumables were used, and media and reagent glass bottles were rinsed with HCl before their use. Radiolabeled siderophores, blocking agents, or any other chemicals were prepared either in sterile water/buffers or filter sterilized before addition to microbial cells.

### 2.2 Analytical methods

To confirm radiolabeling and radiochemical purity (RCP), instant thin layer chromatography (iTLC) was performed using silica gel-impregnated glass microfiber strips (10 cm length) (Agilent Technologies, UK). Later, iTLC strips were scanned using a Raytest Rita-Star TLC scanner (LabLogic, UK) and analyzed using Laura software (LabLogic, UK) or a Cyclone Plus Phosphor Imager (PerkinElmer). Gamma counting was performed using an LKB Wallac 1282 CompuGamma or Perkin Elmer 3470 Wizard2 Gamma Counter.

### 2.3 Microbial strains and growth media

Strains were purchased commercially unless otherwise stated. All strains and their growth media are listed in Table **S2**. The growth conditions were the same for all the bacteria (37 °C) and *Candida* species. *A. fumigatus* was grown at 25-28 °C.

### 2.4 Radiosynthesis of siderophores

^68^GaCl_3_ was eluted following the fractionation approach from a ^68^Ge/^68^Ga generator (Eckert & Ziegler Eurotope GmbH, Berlin, Germany) with 0.1 M ultrapure HCl. Radiolabeling of siderophores was performed according to the published protocol (Table **S1**). The RCP of the ^68^Ga-siderophores was confirmed using iTLC, where 10 % ammonium acetate, 30:70 % water: methanol, or 50 % water:50 % methanol was used as the mobile phase. The stability of gallium-68-labeled siderophores (^68^Ga-siderophores) was determined in assay media by iTLC before performing the assay.

### 2.5 Preparation of microbial cells for the *in vitro* assay

Prior to the *in vitro* uptake assay, the bacterial and *Candida* strains were grown overnight in 5-6 mL of preculture broth (Table **S2**) at 37 °C with shaking at 200 rpm. After overnight incubation, the cells were spun down at 13,000 rpm for 5 min, and pellets were washed with PBS (2 ×).

Iron-depleted bacterial cells were prepared either by (a) preparing iron-depleted stressed cells in minimal media or (b) adding 200 µM 2,2′-dipyridyne (DP) to create iron-depleted medium and later iron-starvation conditions [21]. Briefly, pellets from the initial culture were dissolved in 1 mL assay medium. This suspension (500 µL) was added to 25 mL of assay medium (in a 125 mL flask) for the next round of growth. Cultures were grown at 37 °C with shaking at 200 rpm for 16-21 h to reach the log or stationary phase (considered iron-depleted/iron-starved cells). If needed, the cells were incubated for up to 4 h to obtain the desired log-phase. Iron-repleted cells were prepared in parallel but with the addition of 10 µM FeSO_4_.7H_2_O/FeCl_3_.

*A. fumigatus* cells were prepared as previously published [8]. A spore suspension was prepared from *A. fumigatus* grown on MEA (Malt Extract Agar) agar medium for 5-7 days (25-28 °C). Conidia (1 × 10^6^ per mL) in 25 mL of assay media were inoculated and grown for 18-20 h at 37 °C with 180-200 rpm shaking. These cells are known as iron-depleted *A. fumigatus* cells. Parallel iron-repleted cells were prepared by adding 30 µM FeSO_4_.7H_2_O/FeCl_3_ to the assay medium. The media used for *in vitro* uptake by the different microbes are listed in supplementary Table **S2**.

## 3 Microbial uptake

### 3.1 Iron-dependent

Bacterial and yeast cells prepared from (a and b method) were pelleted down at 4000 × g at 4-10 °C for 15 min. Pellets were washed with PBS (3 ×) using the same conditions described above and dissolved in 2-5 mL of prewarmed (at 37 °C) assay medium (2 mL medium in a 15 mL tube) to obtain the desired amount of CFUs (by OD_600_) measured using a spectrophotometer (SPECTROstar Omega, Germany). For whole-cell uptake, 20-50 µL (0.2-0.6 MBq) of ^68^Ga-labeled siderophores (concentration ranging from 152.32 nM to 3.63 µM) were added to the tube and thoroughly mixed. All tubes were incubated for 45 min at 37°C with shaking at 180 rpm. Control samples (without treatment) were incubated and processed in the same manner.

After 45 min of incubation, a 1 mL sample was transferred into a microfuge tube and centrifuged at 6,000 × g for 5 min. After removing the supernatants in gamma counting tubes, the pellets were washed with ice-cold PBS (3 ×). Both the pellets and supernatants were counted using a gamma counter (γ-counter). The uptake results are expressed as a percentage of the added dose in 10^9^ colony forming units (CFU)/mL viable cells (% AD/10^9^ CFU/mL). The same method was followed for the filter tube assay, and filters (Corning, USA) were counted using a γ-counter.

The uptake of *A. fumigatus* was performed in 96-well filter plates (Millipore, Merck, UK). Then, 180 µL of an overnight (18-20 h) culture was added to a prewetted 96-well filter plate and incubated with 20 µL of ^68^Ga-siderophores at 37 °C. After 45 min of incubation, the plates were filtered and washed with ice-cold Tris (hydroxymethyl)aminomethane (Tris) buffer (3 ×). Filters were collected and counted using a γ-counter.

### 3.2 Blocking assay

For blocking assays, cells were preincubated for 10-20 min at 37 °C with cold Fe-siderophores (uptake blocking gent) at a concentration of 15-20 µM of Fe-DFO-B or Fe-PVD prior to the addition of ^68^Ga-siderophores. Uptake assays were performed as previously described.

### 3.3 Cell viability count

The samples were diluted in PBS to determine the viability of cells after the uptake assays (processed at the same time as the assay sample or stored at 4 °C until processing). One hundred microliters of desired dilutions of the sample (10^-6^, 10^-5^, 10^-4^, 10^-3^, 10^-2^) were spread onto agar media (Table **S2**) and incubated at 37°C overnight in triplicate. Alternatively, 10 µL of the desired dilution of the sample was spotted in triplicate. Cell viability was expressed as CFU/mL. Control samples were also incubated for cell viability.

### 3.4 Statistical data analysis

All uptake data were processed using Microsoft Office Excel 2019. All graphs are depicted with error bars corresponding to the standard deviation and were created using GraphPad Prism 10. P values were calculated using the ‘unpaired t-test’ for statistical significance on GraphPad Prism 10, with a P value equal to or less than 0.05 considered as statistically significant (*P ≤ 0.05, **P ≤ 0.01,***P ≤ 0.001,****P ≤ 0.0001, and ns (non-significant) when P > 0.05).

## 4 Results and discussion

### 4.1 Validation of ^68^Ga-siderophore uptake

In this study, we radiolabeled selected siderophores according to the published protocols with gallium-68 (Table **S1**). Enterobactin (ENT), produced by *E. coli,* and triacetylfusarinine C (TAFC), produced by *A. fumigatus,* were used for the uptake validation in *E. coli* and *A. fumigatus*, respectively.

Desferrioxamine B (DFO-B) as xenosiderophore, was evaluated in bacterial and fungal species to determine its uptake specificity among selected pathogens. For the DFO-B evaluation, ferrichrome (FCH) was used as a positive control for bacterial and fungal cells, and coprogen (COP) was used as a negative control for *A. fumigatus*.

We validated and optimized the *in vitro* uptake of gallium-68-labeled ENT ([^68^Ga]Ga-ENT) by *Escherichia coli* considering important microbiological and experimental factors. Our findings showed that the medium with the minimum nutrients for microbial growth produced consistent uptake results. This is because, in minimal media, microbes prepare their metabolism for survival rather than rapid growth. Moreover, preculturing in the assay medium before the original uptake assay provided an adaptation time for the tested strains [22]. The iron-depleted condition in minimal medium 9 (MM9) showed a higher uptake of [^68^Ga]Ga-ENT than the iron-replete condition.

Although siderophores are produced and excreted during the exponential phase of bacterial growth, siderophore production is typically higher during the stationary phase [23]. When the growth phase (mid log vs. stationary) was compared, uptake was higher in stationary cells expressed as 10^9^ CFU/mL (% AD/10^9^ CFU/mL) (supplementary Figure **S1**). This is because mid log phase *E. coli* cells demonstrated higher viability than early stationary phase *E. coli* cells as mid log phase bacteria are the most rapidly growing cells with high metabolic activity. The reduction in cell viability in the early stationary phase cells could be due to the stress condition in the stationary phase under iron-depleted conditions.

**Fig. 1:**
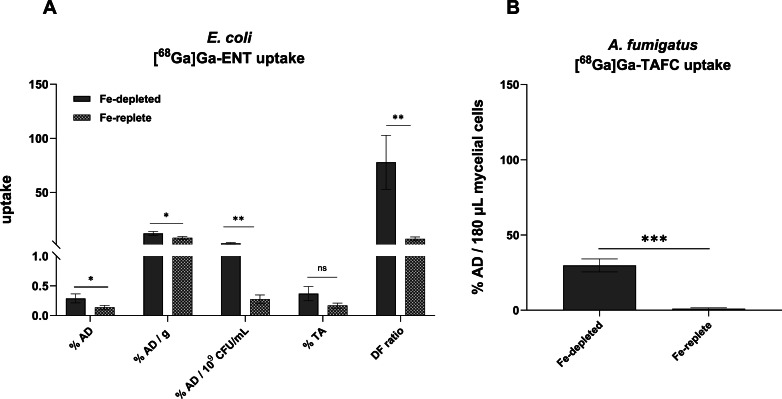
Validation of *in vitro* uptake of ^68^Ga-siderophores in microbial cells. (A) Uptake of [^68^Ga]Ga-ENT by *E. coli* expressed using different metrics. Uptake performed in early stationary phase *E. coli* cells after incubation with [^68^Ga]Ga-ENT for 45 min at 37°C with shaking under iron-depleted and iron-replete (adding 10 µM FeCl_3_) conditions. **‘% AD/10^9^ CFU/mL’** showed higher significance when compared between iron-depleted and iron-replete conditions considering the total viable counts. (B) Uptake of [^68^Ga]Ga-TAFC by *A. fumigatus* in a 96-well filter plate. Uptake was performed by incubating 180 µL of mycelial cells with [^68^Ga]Ga-TAFC for 45 min at 37°C with shaking under iron-depleted and iron-replete (30 µM FeCl_3_) conditions. Error bars indicate standard deviations from three individual samples. The unpaired t-test for comparison among test groups showed significant differences (a P value of <0.05 expressed as ‘*’). [% AD: ‘% Added Dose’, considers activity in the cell pellets; ‘% AD/g of pellet’ considers the total weight of the pellets; ‘% AD/10^9^ CFU/mL’ expresses activity in the cell pellets in 10^9^ cells; % TA: ‘% Total Activity’, expresses activity there is in the cell pellets compared to the supernatant; DF: ‘Distribution ratio’, expresses the ratio of intracellular to the extracellular activity considering total volume of viable cells; CFU = colony forming units.].

A comparison of five expression methods for uptake results demonstrated that % AD/10^9^ CFU/mL represented the most reliable approach, considering actual radioactivity in the sample (% AD) and the total number of viable cells after incubation with [^68^Ga]Ga-ENT (Fig. 1A). Statistical analysis showed that the difference between iron-depleted and iron-replete cells was more significant when the results were expressed as ‘% AD/10^9^ CFU/mL’. Other expression methods, for instance, ‘% of total activity in the pellet (% TA)’ [12,24] gave an idea of what percentage of activity was present in the bacterial cells compared to the assay medium. The drawback of the % AD/g of pellets [10,14] is that it considers the total weight of the pellet without knowing the initial inoculum and viability after the uptake. % AD, % TA and % AD/g all may have the drawback of measuring activity containing both live and dead cells. The distribution ratio of intracellular to extracellular radioactivity (DR) which considers total viable cells, yielded estimated results because microbial cell size can vary with different growth conditions and stages [25].

Additionally, the uptake assay was performed on a small scale (1 mL microfuge tube with 500-1000 µL cells and filter tubes) (not shown here). All showed adequate cell washing with buffers is crucial to wash off unbound radiotracers. Otherwise, there will be a false-high uptake of radiotracers.

On the other hand, 96-well filter plate experiments [8] for fungal uptake assays with [^68^Ga]Ga-TAFC (Fig. 1B) revealed technical challenges with *Aspergillus fumigatus* in terms of adding a fixed number of mycelial cells to each well, low binding of mycelial cells to the filter and punching out the filter after drying. An automated punching machine could resolve the latter issue. More replicates (>3) are recommended to reduce the variability of the results. The results reporting method for fungal uptake in the literature includes % AD and % AD/microgram of proteins. It is essential to maintain the reproducible methodology and defined volume of cell inoculum while preparing the preculture. In our study, we found that [^68^Ga]Ga-TAFC uptake, expressed as % AD/180 µL of mycelial cells, was higher in iron-depleted conditions (Fig. 1B).

### 4.2 [^68^Ga]Ga-DFO-B uptake by bacterial and fungal cells

**Fig. 2:**
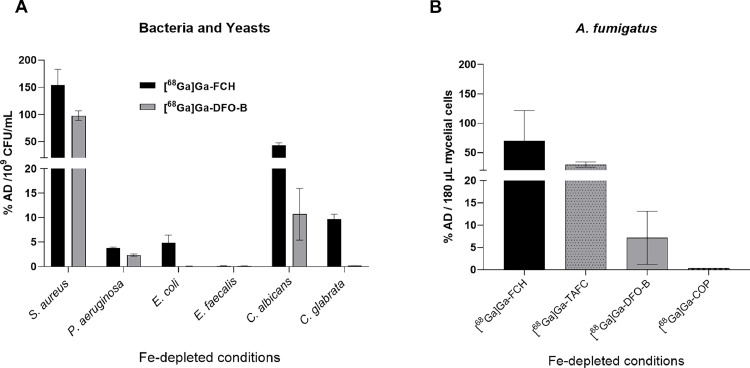
[^68^Ga]Ga-DFO-B uptake in bacterial and fungal cells. (A) Bacterial and yeast cells were incubated with [^68^Ga]Ga-DFO-B and [^68^Ga]Ga-FCH for 45 min at 37°C with shaking under iron-depleted conditions. Uptake was expressed as ‘% AD/10^9^ CFU/mL’ and showed higher uptake of [^68^Ga]Ga-DFO-B by *S. aureus* compared to other bacteria and yeast. (B) Uptake of [^68^Ga]Ga-DFO-B by *A. fumigatus* in a 96-well filter plate comparing with other ^68^Ga-siderophores. Uptake was performed by incubating 180 µL of mycelial cells with [^68^Ga]Ga-DFO-B for 45 min at 37°C with shaking under iron-depleted conditions and expressed as ‘% AD/180 µL mycelial cells. Results showed poor uptake of [^68^Ga]Ga-DFO-B by *A. fumigatus*. Error bars indicate standard deviations from three individual samples. [% AD: ‘% Added Dose’].

After initial validation studies, we evaluated the clinically approved siderophore DFO-B for its *in vitro* uptake specificity among bacterial and fungal cells compared with ferrichrome (FCH) (Fig. 2A-B). Both DFO-B and FCH are xenosiderophores for our selected microbial species. Moreover, FCH is known to be utilized by many bacterial and fungal cells, and in this study, we used FCH as a positive control. We observed a high uptake of [^68^Ga]Ga-DFO-B by *S. aureus.* Published studies also showed higher uptake of this radiotracer by *S. aureus* strains compared to coagulase-negative *S. agalactica* [14]. However, those studies did not include viability data. Uptake of [^68^Ga]Ga-DFO-B by *P. aeruginosa* was also observed as expected [14]. Although *E. coli* possesses the periplasmic siderophore binding protein FhuD for ferrioxamine B [26], it did not show [^68^Ga]Ga-DFO-B uptake in either *in vitro* or *in vivo* infection models [14]. Our study also showed poor uptake of [^68^Ga]Ga-DFO-B by *E. coli*. The literature shows that not all *E. faecalis* strains can utilize xenosiderophores [[27], [28], [29]]. *E. faecalis* showed no uptake of the ^68^Ga-siderophores used in this study. *C. albicans* showed low uptake of [^68^Ga]Ga-DFO-B compared to [^68^Ga]Ga-FCH, however, *C. glabrata* showed no uptake of [^68^Ga]Ga-DFO-B. Though this uptake was expressed as % AD/10^9^ CFU/mL, the level of uptake is not actually comparable with bacterial uptake due to the physical and molecular differences between bacterial and yeast cells. Due to the technical challenges faced with *A. fumigatus* uptake validation assays, we used [^68^Ga]Ga-COP and [^68^Ga]Ga-TAFC as negative and positive controls, respectively, as the literature shows poor uptake of [^68^Ga]Ga-COP by *A. fumigatus* [30]. [^68^Ga]Ga-DFO-B was poorly taken up by *A. fumigatus* compared to the positive control. Our uptake studies in an iron-depleted assay medium were expected to have low pH, which resulted in poor or no uptake of [^68^Ga]Ga-DFO-B by *A. fumigatus,* consistent with previously published studies [8]. However, a recent study showed that the [^68^Ga]Ga-DFO-B uptake efficacy by *A. fumigatus* was low at acidic pH and increased at neutral and alkaline pH owing to its chemical structure and charge status at different pH values [15]. This pH-dependent uptake was supported by the presence of ferrioxamine B membrane transporter genes in *A. fumigatus* [30,31]. It demonstrated that the structure, charge of the siderophores, and the pH of the media are also important for the uptake by microbial cells.

The uptake specificity of [^68^Ga]Ga-DFO-B was determined by adding the uptake-blocking agent Fe-DFO-B and Fe-PVD (for *P. aeruginosa*) at excess concentrations (supplementary Figure **S2**). It was determined that excess concentrations (× 50-100, the concentration) were needed to block uptake in the assessed bacterial strains. This time, we also evaluated uptake by *S. epidermidis* since *S. epidermidis* is one of the major pathogens for VGEI, and it showed the highest uptake among all the strains.

### 4.3 Effect of [^68^Ga]Ga-DFO-B uptake on cell viability

The viability assay showed minimal or no effect of [^68^Ga]Ga-DFO-B on the bacterial strains, except for *S. epidermidis* (supplementary Figure **S3**). The 100-fold reduction in viability and subsequent 10-fold recovery with the addition of excess Fe-DFO-B in *S. epidermidis* is due to the bacteriostatic effect of DFO-B on coagulase-negative staphylococci [32,33]. Although glucose as the sole carbon source in Yeast Extract Peptone Glucose (YPG) medium could induce stress conditions in bacteria, 200 µM DP was added to the assay medium to confirm the iron-stressed condition [17], which could lead to further reduction in the viability of *S. epidermidis*. This was later supported by a comparison of [^68^Ga]Ga-DFO-B uptake by *S. aureus* in YPG ± 200 µM DP medium. Without 200 µM DP, there was a 1.4-fold reduction in cell viability; however, 200 µM DP treatment resulted in a ∼6-fold reduction (supplementary Figure **S4**). This inhibition is likely to occur because of iron deprivation in cells that could otherwise be used in metabolism [33,34].

### 4.4 [^68^Ga]Ga-DFO-B uptake by *S. aureus* with low CFU/mL

To develop a diagnostic method for a particular infection, sensitivity to the *in vivo* microbial load (CFU/mL) is crucial. Early infection diagnosis refers to an acute infection with a low to high number of pathogens, depending on the infection site and nature of the pathogen. A preclinical study showed that the threshold for bacteria to cause graft infection in >50 % of aortic grafts in canine models could be as low as 10^2^ for *P. aeruginosa* [35]. However, for ventilator-associated pneumonia, there is a range of quantitative cultures from 10^3^ to 10^6^ CFU/mL [36]. In immunocompromised individuals, even a small number of pathogens is likely to cause infection. In this study, we showed that a minimum of 10^2^ CFU/mL of *S. aureus* cells were able to demonstrate detectable radioactivity of [^68^Ga]Ga-DFO-B in raw results. However, uptake was very low when expressed as % AD (supplementary Figure **S5**), and total viable count was not possible due to the use of filter tubes for the experiment. Therefore, the data were not normalized to % AD/10^9^ CFU/mL. It is speculated that the high sensitivity of PET imaging can localize pathogens, even at low levels *in vivo* [10], unless the effect of tracers on the pathogen is inhibitory [37,38].

In summary, *in vitro* short-term uptake assays can provide excellent preclinical data to determine the uptake specificity of ^68^Ga-siderophores among clinically relevant pathogens. Therefore, published results should provide reproducible *in vitro* assays with positive and negative controls and microbiological and technical information. Although *in vitro* uptake assays do not provide structural binding information, the use of blocking and competing compounds can validate tracer specificity in microbial strains. The microbiological load, viability, and inhibitory effects of siderophores on the tested strains should be reported. Thorough literature searches are crucial, as xenosiderophore utilization is critical because of intra- and interspecies (polymicrobial infection) variations in siderophore transport induction under iron-depleted conditions [[39], [40], [41], [42]]. It also gives the opportunity to develop both broad-spectrum and narrow-spectrum infection-specific radiotracers, which would then be used in combination or sequentially. *In vitro*/*ex vivo* studies with co-culture can provide further specificity before forwarding to *in vivo* infection model studies. Evaluation of potential radiotracers in both drug-sensitive and drug-resistant clinical strains should be performed. Moreover, the absence of siderophore-binding receptors/proteins in bacteria of the same species but belonging to different strains could lead to false-negative results. However, large-scale gene sequencing of clinical strains is needed to identify whether specific genes or variations allow the uptake of xenosiderophores. This could be crucial for the proper interpretation of ^68^Ga-siderophore imaging studies. Chronic infections often lead to biofilm formation [[43], [44], [45]]. Therefore, it is important to consider the type and status of the infection when developing new diagnostic tools. It would be useful to develop and evaluate siderophore radiotracers in *in vivo* models, in which pathogens are quiescent within biofilms. To date, the ability of radiotracers to penetrate biofilms has been inadequately studied.

## 5 Conclusion

Undoubtedly, ^68^Ga-siderophores demand more preclinical research to translate as a diagnostic tool to clinics. Our *in vitro* validation with [^68^Ga]Ga-ENT shows uptake was higher in minimal assay medium by stationary phase *E. coli* cells. When compared with different approaches of expressing uptake results, % AD/ 10^9^ CFU/mL (viable cells) offers a way of comparing uptake results among different microbial species. Clinically approved siderophore DFO-B labeled with gallium-68 shows high to moderate uptake by *S. aureus, S. epidermidis, P. aeruginosa*, and *C. albicans*, and minimal to no uptake by *E. coli, A. fumigatus, E. faecalis*, and *C. glabrata*. Furthermore, the cell viability of all the tested bacterial strains was minimally affected in the presence of [^68^Ga]Ga-DFO-B during the 45-minute short-term assay period, except for *S. epidermidis*.

## CRediT authorship contribution statement

**Asma Akter:** Writing – review & editing, Writing – original draft, Methodology, Investigation, Conceptualization. **Margaret S. Cooper:** Writing – review & editing, Methodology. **Afnan M.F. Darwesh:** Writing – review & editing, Methodology, Investigation. **Robert C. Hider:** Writing – review & editing, Conceptualization. **Philip J. Blower:** Writing – review & editing, Conceptualization. **Nicholas M. Price:** Writing – review & editing, Resources. **Oliver Lyons:** Writing – review & editing. **Silke Schelenz:** Writing – review & editing, Resources. **Varun Mehra:** Writing – review & editing, Investigation. **Vincenzo Abbate:** Writing – review & editing, Supervision, Investigation, Conceptualization.

## Declaration of competing interest

There are no competing interests from any authors.

## Appendix Supplementary materials

Image, application 1
