# Supplementary material for: Radiotracers for in situ infection imaging: Experimental considerations for in vitro microbial uptake of gallium-68-labeled siderophores
Source: Diagn Microbiol Infect Dis. 2024 Dec;110(4):116522. doi: 10.1016/j.diagmicrobio.2024.116522 (PMC11846779; doi:10.1016/j.diagmicrobio.2024.116522)
Supplement: Supplementary file 1 [file mmc1.docx]

**Radiotracers for *in situ* infection imaging: Experimental considerations for *in vitro* microbial uptake of gallium-68-labeled siderophores**

**Table S1.** List of siderophores and their producer and ^68^Ga radiolabeling methods in this study.

| **Siderophore/source** | **Producer** | **^68^Ga Radiolabeling Method*** |
| --- | --- | --- |
| Enterobactin (ENT), Sigma‒Aldrich (UK) | *Escherichia coli*  (Additionally, *Salmonella typhimurium*, *Shigella* spp., *Klebsiella* spp. and *Enterobacter* spp.) | Acetate buffer at room temperature for 10-15 min [1] |
| Triacetylfusarinine C (TAFC), Biophore Research Products (Germany) | *A. fumigatus, A. nidulans, Fusarium graminearum* | Acetate buffer at room temperature for 10-15 min [2] |
| Ferrichrome (FCH), Sigma‒Aldrich (UK) | *Aspergillus niger, A. quadricinctus, Penicillium parvum, Ustilago sphaerogena, U. maydis, Neovossia indica, Trichophyton mentagrophytes, Schizosaccharomyces pombae* | Acetate buffer at room temperature for 10-15 min [3] |
| Coprogen (COP), Biophore Research Products (Germany) | *Pseudomonas chrysogenum, Neurospora crassa, A. terrus, Cochilibolus heterostrophus, Magnaporthe grisea, Colletotrichum graminicola* | Acetate buffer at room temperature for 10-15 min [3] |
| Desferrioxamine B (DFO-B), Sigma‒Aldrich (UK) | *Streptomyces pilosus*  (Additionally, *Arthrobacter simplex*, *Erwinia herbicola*) | Acetate buffer at room temperature for 10-15 min [4] |
| Pyoverdine (PVD), Sigma-Aldrich (UK) | *P. aeruginosa* | - |

* After the reaction, extra sodium acetate was added to increase the pH to 6-7, if required. Their radiochemical purity (RCP) of ≥ 95% was confirmed using instant thin-layer chromatography (iTLC).

**Table S2**. Microbial strains, growth and assay media were used in this study.

| **Bacteria ​** | **Growth media** | **Uptake assay media**​ | **Reference**​ |
| --- | --- | --- | --- |
| *Escherichia coli*  NCIMB 10218 | Luria–Bertani (Lenox) agar/broth | Minimal Medium 9 (MM9)*  ​ | [5] |
| *Pseudomonas aeruginosa*  NCTC 10662 | Trypticase Soy agar/broth | Davis minimal Medium (Sigma-Aldrich) | This study |
| *Staphylococcus aureus* ​  NCTC 6571 | Trypticase Soy agar/broth | Yeast Extract-Peptone-Glucose (YPG) Medium/ RPMI + 1 % (w/v) casamino acid* | [6], [7] |
| *Staphylococcus epidermidis* ​  NCIMB 8853 | Trypticase Soy agar/broth | YPG Medium**​ | [6] |
| *Enterococcus faecalis*  ATCC 29212 ​ | Brain Heart Infusion agar/broth | N Medium* ​ | [8] |
| *Candida albicans*  ATCC 90028 | Sabours Dextrose (SD), YPD Agar/broth | Glucose minimal medium (GMM)* | [9] |
| *Candida glabrata*  ATCC 90030 | SD, YPD Agar/broth | YPG Medium**​/GMM* | [6], [9] |
| *Aspergillus fumigatus*  ATCC 46640 | Malt extract agar (MEA) or SDA medium | Aspergillus minimal medium (AMM)* | [10] |

*10 - 30 µM FeSO_4_.7H_2_O/FeCl_3_ added to obtain Iron-replete conditions

**200 µM 2,2’-dipyridyne (DP) added to obtain Iron-depleted conditions

*E. faecalis* was generously provided by Prof. Nicholas Price, Department of Infectious Diseases, Guy’s & St Thomas’ NHS Foundation Trust, London, UK. *C. albicans*, *C. glabrata*, and *A. fumigatus* were kindly provided by Dr. Silke Schelenz, KCH Clinical Lead Infection Sciences, King’s College Hospital, London, UK.

**Figure S1**. Uptake of [^68^Ga]Ga-ENT by early stationary versus mid log phase *E. coli* cells in MM9 medium (iron-depleted conditions). AD: Added dose. Early stationary cells showed higher uptake than mid log cells when expressed as % AD/10^9^ CFU/mL. Error bars indicate standard deviations from three individual samples. The unpaired t-test for comparison among two test groups showed significant differences (a P value of <0.05 expressed as ‘*’).

**Figure S2**. *In vitro* uptake specificity of ^68^Ga-labeled desferrioxamine B ([^68^Ga]-Ga-DFO-B) by early stationary bacterial cells under iron-depleted conditions. Uptake of [^68^Ga]Ga-DFO-B by *S. aureus*, *S. epidermidis*, *E. faecalis*, *E. coli* and *P. aeruginosa* after 45 min incubation (±Fe-DFO-B and Fe-PVD, ~20 µM as blocking agent). Error bars indicate standard deviations from three individual samples. Blocking agents were able to block the uptake of [^68^Ga]-Ga-DFO-B in *S. epidermidis,* *S. aureus* and *P. aeruginosa* demonstrating the uptake specificity in these strains.

Blocking agent

[^68^Ga]Ga-DFO-B

[^68^Ga]Ga-DFO-B + Blocking agent

**Figure S3**. Determination of total viable cell counts after incubation with [^68^Ga]Ga-DFO-B under iron-depleted conditions. 2,2’-Dipyridyne (DP) (200 µM) was added for [^68^Ga]Ga-DFO-B uptake studies in *S. epidermidis*. Error bars indicate standard deviations from three individual samples. The results showed no to minimal effect of [^68^Ga]Ga-DFO-B on the viability of *E. coli*, *P. aeruginosa*, *S. aureus* and *E. faecalis*. However, cell viability was reduced up to 100-fold for *S. epidermidis*.

**Figure S4**. Effect of 2,2’-dipyridyne (DP) (200 µM) on the cell viability of *S. aureus* under iron-depleted conditions. Error bars indicate standard deviations from three individual samples. The results show that the addition of DP reduced cell viability during [^68^Ga]Ga-DFO-B uptake. The unpaired t-test for comparison among two test groups showed significant differences (a P value of <0.05 expressed as ‘*’).

**Figure S5.** Uptake of [^68^Ga]Ga-DFO-B by *S. aureus* with different microbial loads (CFU/mL) in iron-depleted conditions. Error bars indicate standard deviations from three individual samples.

**References**

[1] K. Bendova et al., “[^68^Ga]Ga-Ornibactin for Burkholderia cepacia complex Infection Imaging Using Positron Emission Tomography,” J Med Chem, vol. 66, no. 11, 2023, doi: 10.1021/acs.jmedchem.3c00469.

[2] M. Petrik et al., “Preclinical evaluation of two ^68^Ga-siderophores as potential radiopharmaceuticals for Aspergillus fumigatus infection imaging,” Eur J Nucl Med Mol Imaging, vol. 39, no. 7, 2012, doi: 10.1007/s00259-012-2110-3.

[3] M. Petrik, H. Haas, M. Schrettl, A. Helbok, M. Blatzer, and C. Decristoforo, “In vitro and in vivo evaluation of selected ^68^Ga-siderophores for infection imaging,” Nucl Med Biol, vol. 39, no. 3, 2012, doi: 10.1016/j.nucmedbio.2011.09.012.

[4] Afnan M. F. Darwesh. Gallium-Essential Applications of Gallium-67 and Gallium-68. . King’s College London , 2021.

[5] H. Abdul-Tehrani et al., “Ferritin mutants of Escherichia coli are iron deficient and growth impaired, and fur mutants are iron deficient,” J Bacteriol, vol. 181, no. 5, 1999, doi: 10.1128/jb.181.5.1415-1428.1999.

[6] M. Petrik et al., “Imaging of Pseudomonas aeruginosa infection with Ga-68 labelled pyoverdine for positron emission tomography,” Sci Rep, vol. 8, no. 1, 2018, doi: 10.1038/s41598-018-33895-w.

[7] F. C. Beasley et al., “Characterization of staphyloferrin A biosynthetic and transport mutants in Staphylococcus aureus,” Mol Microbiol, vol. 72, no. 4, pp. 947–963, May 2009, doi: 10.1111/j.1365-2958.2009.06698.x.

[8] M. Latorre et al., “The role of fur in the transcriptional and Iron homeostatic response of Enterococcus faecalis,” Front Microbiol, vol. 9, no. JUL, 2018, doi: 10.3389/fmicb.2018.01580.

[9] C. J. Hu, C. Bai, X. De Zheng, Y. M. Wang, and Y. Wang, “Characterization and functional analysis of the siderophore-iron transporter CaArn1p in Candida albicans,” Journal of Biological Chemistry, vol. 277, no. 34, 2002, doi: 10.1074/jbc.M204545200.

[10] M. Schrettl et al., “SreA-mediated iron regulation in Aspergillus fumigatus,” Mol Microbiol, vol. 70, no. 1, 2008, doi: 10.1111/j.1365-2958.2008.06376.x.
